# A Statement in Relation to the United States Naval Medical Corps

**Published:** 1848

**Authors:** 


### ARTICLE VII.

### *A Statement in relation to the United States Naval Medical Corps.*

Believing it to be in accordance with the objects of this Association, we beg leave to lay before it certain facts in relation to that portion of the medical profession associated with the naval service, and which we have the honor to represent in the present assembly.

From the large number of persons who appear before the Naval Medical Examining Boards, and from the small proportion who are successful, it has been intimated that influence, independent of qualification, is essential to success. Such an impression is of course adverse to any efforts the Government Boards may make to elevate the profession, and its correction is due not only to the Boards, but to the profession at large.

The aid of influential friends is not necessary even to obtain a permission to appear before the Boards, but it is granted to all within the prescribed ages, disposed to enter the field of competition, without question as to political alliance or social position, but with the understanding that a limited number of appointments is to be made, and those alone can be successful who are found to be best qualified.

The Boards vary as to their individual composition: the selection of their members being made from such medical officers, sufficiently old in the service, as the public interests permit to be taken, from time to time, from other duties; and they are convened under a precept containing the following injunction:

"The attention of the Board will be directed to moral character, as well as to scientific and other attainments; and it will be its duty to make the examination full, minute, and rigid."

From the limited number of medical officers, and the want of allowance for inefficient members in their corps, it becomes both the duty and interest of Boards to select only such as are physically competent to their duties: hence, many may be set aside for essential reasons other than professional incompetency. Again, duties in the naval medical service require a practical knowledge upon certain branches, not attained by many otherwise well informed in their profession. For instance—medical officers on board ship are often required to be their own apothecaries; and in foreign countries to select their medicines, when they must depend upon their practical acquaintance with drugs to select those of good quality. Deficiency in such knowledge excludes many.

For admission into the naval service, a fair preliminary education, and a knowledge of the branches strictly professional, are the nominal requirements, but, from the amount of competition, higher attainments are necessary to secure success. Professional acquirements being equal, those persons would be selected who possessed in addition a knowledge of collateral sciences and of languages: relative position, a matter of importance, being decided according to the amount of professional and general information of the successful candidates. The mode of examination is as follows: in the first place the applicant is required to reply to the following

"CIRCULAR TO CANDIDATES.

"For the information of the Board, you will state, in your own handwriting, the place of your birth, your age, the State of which you are a citizen, and the names of the institutions in which your general education has been acquired. If a graduate of arts, please state from what college you received your diploma. Besides English, what languages have you studied? If you have studied natural history, please state what branches.

"State the name of your medical preceptor, and the time devoted by you to the study of medicine. If a graduate, of what institution? What period of time have you devoted to practical anatomy or dissection? What opportunities have you had to witness the practice of medicine and surgery?—What opportunity, if any, have you had to become acquainted with pharmacy and the physical properties of drugs?

"You will state, on your honor, whether you are obnoxious to any hereditary disease whatever; especially whether any of your immdiate family has suffered from pulmonary disease, epilepsy, insanity, or paralysis: and you will also state whether your general health is good, and whether you are free from constitutional disease and local affection, such as hernia, &c. You will furnish satisfactory evidence that your moral and social habits and character are good."

The reply to this circular not only furnishes the Board with the information asked by it, but gives some information as to the facility of composition and knowledge of orthography possessed by the candidates; and upon these points many fail. If the reply is satisfactory, the candidate is then furnished with a single sheet of foolscap, and a professional subject, upon which he is required to write in an apartment adjoining that of the Board's session.

The examinations upon surgery, materia medica and pharmacy, are partially practical; the candidate being required to apply various dressings and apparatus, to designate the medicines and preparations set before him unlabelled, and to write and compound prescriptions.

Before a recent Board, one gentleman defined a lotion to be "a kind of application," and an evaporating lotion "one which does not evaporate." Another confessed his ignorance of the freezing and boiling points of water, and contended that knowledge upon such subjects was useless. One candidate determined castor oil to be the "oil of castor, an animal." Another located the solar plexus in the sole of the foot. All these were graduates.

The foregoing facts will, we think, sufficiently account for a large rejection, without invoking the inference of political disqualification.

In contradiction to the idea of the power of other influences than those of professional qualification, the results of the examinations show those to be most successful whose energies have been developed and faculties strengthened under a continued struggle with necessity and limited means; while too many, aided by influential friends, possessed of ample means and all the facilities these control, have been found unfaithful servants in the improvement of the talents placed at their disposal.

The medical corps of the navy, in its insulated position, has had devolved upon it the unpleasant responsibility of advocating, against contending influences, its own interests, and the claims of the medical profession to respectability, assailed through the corps.

Until recently, it has been left as a portion of a military body, without any defined position, being dependent for its social standing, and the respect awarded its members, upon the individual and peculiar views of military superiors and associates: these were too frequently annoying to the medical officers and derogatory to the profession of which they are members.

After the long-continued and arduous efforts of the medical officers, the executive became assured of the injustice of their position, and by a general order issued August, 1846, they were assimilated in rank with medium classes of their military brethren; and certainly not placed in a higher comparative position than the profession of medicine can justly claim. The "order" merely defines position and confers no military authority. Opposition has, however, been made to this arrangement by a portion of the line, and, at its instigation, an inquiry instituted upon the floor of Congress, with the view, it is believed, to an attempt to revoke the position assigned medical officers in the navy.

Such is the existing state of interests, which we feel it our duty, through the medium of this Association, to bring to the notice of the profession of which we form a small and isolated portion.

WM. MAXWELL WOOD, *Surg. U.S.N.*,
NINIAN PINKNEY, *Surg. U.S.N.*,
*Delegates to the Nat. Med. Association,*
*from the Naval Med. Corps.*

—*Trans. Am. Med. Association.*

